# Abscisic Acid Mediates Drought-Enhanced Rhizosheath Formation in Tomato

**DOI:** 10.3389/fpls.2021.658787

**Published:** 2021-07-23

**Authors:** Joseph K. Karanja, Mehtab Muhammad Aslam, Zhang Qian, Richard Yankey, Ian C. Dodd, Xu Weifeng

**Affiliations:** ^1^Center for Plant Water-Use and Nutrition Regulation and College of Life Sciences, Joint International Research Laboratory of Water and Nutrient in Cops, Fujian Agriculture and Forestry University, Fuzhou, China; ^2^China National Engineering Research Center of Juncao Technology, Fujian Agriculture and Forestry University, Fuzhou, China; ^3^The Lancaster Environment Centre, Lancaster University, Lancaster, United Kingdom

**Keywords:** ABA, drought stress, transcriptome analysis, rhizosheath formation, tomato

## Abstract

The rhizosheath, commonly defined as soil adhering to the root surface, may confer drought tolerance in various crop species by enhancing access to water and nutrients under drying stress conditions. Since the role of phytohormones in establishing this trait remains largely unexplored, we investigated the role of ABA in rhizosheath formation of wild-type (WT) and ABA-deficient (*notabilis, not*) tomatoes. Both genotypes had similar rhizosheath weight, root length, and root ABA concentration in well-watered soil. Drying stress treatment decreased root length similarly in both genotypes, but substantially increased root ABA concentration and rhizosheath weight of WT plants, indicating an important role for ABA in rhizosheath formation. Neither genotype nor drying stress treatment affected root hair length, but drying stress treatment decreased root hair density of *not*. Under drying stress conditions, root hair length was positively correlated with rhizosheath weight in both genotypes, while root hair density was positively correlated with rhizosheath weight in well-watered *not* plants. Root transcriptome analysis revealed that drought stress increased the expression of ABA-responsive transcription factors, such as AP2-like ER TF, alongside other drought-regulatory genes associated with ABA (ABA 8′-hydroxylase and protein phosphatase 2C). Thus, root ABA status modulated the expression of specific gene expression pathways. Taken together, drought-induced rhizosheath enhancement was ABA-dependent, but independent of root hair length.

## Introduction

Drought conditions often limit plant growth and yields in agricultural systems (Mahalingam, 2015; Anjum et al., 2017; Hussain et al., 2018), thereby threatening the food security of the ever-surging global population (Long et al., 2015). The frequency and severity of drought are predicted to increase with climate change (Battisti and Naylor, 2009; Mach et al., 2019). Drying stress modifies root system architecture (RSA) to increase water uptake, potentially enhancing plant growth and yields (Jeong et al., 2013; Uga et al., 2013). RSA describes the three-dimensional organization of different root types (e.g., primary and laterals) in the soil (Lynch, 1995; Ning et al., 2012; Smith and De Smet, 2012). Deeper roots and increased root density can enhance plant growth under water deficit conditions by enhancing water and nutrient acquisition from the heterogeneous soil environment (Lynch et al., 2014; Zhan et al., 2015). Plant hormones play a crucial role in root growth and development (Davies, 2010). Among classical phytohormones, abscisic acid (ABA) has been widely considered as a stress hormone, and its role in regulating plant drought stress responses has been extensively studied (Schachtman and Goodger, 2008; Cutler et al., 2010). Drying stress treatment stimulates ABA accumulation, which plays an important role in maintaining root elongation (Saab et al., 1990; Giuliani et al., 2005) as well as root hair formation and elongation in *Arabidopsis* and crop plants (Chen et al., 2006; Xu et al., 2013).

At a microscopic scale, root architecture includes root hairs, tubular extensions of root epidermal cells that emerge behind the root elongation zone (Peterson and Farquhar, 1996; Richardson et al., 2009; Pereg and McMillan, 2015). Root hairs account for 70–90% of the total root surface area, and increase the soil volume from which roots can acquire resources (Smith and De Smet, 2012; Kwasniewski et al., 2016). Enhanced root hair formation is one of many potential mechanisms by which plants can enhance tolerance to soil water deficits (White et al., 2013a,b). Although WT and root hairless mutants had similar physiological and agronomic responses when grown with adequate water availability, WT plants had better water status and lower foliar ABA concentration under soil water deficit conditions (Marin et al., 2020), indicating the adaptive significance of root hairs.

The rhizosheath is defined as soil adhering to roots upon excavation, and it may enhance water status of root tissues as the soil dries (George et al., 2014). Rhizosheath formation is influenced by several factors, such as root and/or microbial mucilage (Watt et al., 1994; McCully, 1999; Carminati et al., 2017), microbial activities (Hanna et al., 2013), soil physicochemical properties (Haling et al., 2014), and root hair traits (Haling et al., 2010). Long root hairs have been associated with larger rhizosheaths in barley and wheat genotypes (Haling et al., 2014; Delhaize et al., 2015), but few studies have described the genetics of rhizosheath formation under water deficit conditions (George et al., 2014), or determined the involvement of plant hormones in rhizosheath formation.

Most attention has focused on the role of ABA in mediating rhizosheath formation by affecting root hair elongation. While 1 μM exogenous ABA inhibits Arabidopsis root hair growth *in vitro* through transcriptional regulation (Rymen et al., 2017), similar ABA concentrations stimulated root hair elongation of hydroponically grown rice seedlings (Wang et al., 2017). While attenuating ABA signaling by overexpressing *OsABIL2* in rice produced shorter root hairs (Wang et al., 2017), the root hair phenotype of ABA-deficient mutants has attracted little attention. When grown hydroponically, the ABA deficient *Az34* barley mutant had a shorter root hair zone than WT plants (Sharipova et al., 2016), which might explain its limited rhizosheath formation independent of soil water availability (Zhang et al., 2021). Conversely, barley genotypes that either had or lacked root hairs (and thus differed in rhizosheath formation (George et al., 2014) had similar root xylem ABA concentrations when young vegetative plants were grown under drying stress conditions (Dodd and Diatloff, 2016). However, the involvement of ABA in rhizosheath formation remains obscure, especially for dicotyledonous crops such as tomato.

Other studies have identified some potential genes (e.g., glutamate receptor GLR3.1) that may explain the genetics of barley rhizosheath (George et al., 2014) possibly by enhancing root growth (Li et al., 2006; Gamuyao et al., 2012). Many genes encoding transcription factors (TFs), either induced by ABA treatment or ABA independent, have been identified in roots responsive to drying stress (Recchia et al., 2013; Janiak et al., 2016). For example, a gene encoding dehydration-responsive element-binding1 (DREB 1) belonging to the wider family APETALA2 ethylene-responsive element-binding TFs was upregulated in maize roots under water stress conditions (Liu et al., 2013). Hence, water deficit stress perception, signaling, and regulatory pathways controlling the expression of stress-responsive genes mediate root growth, which may increase soil moisture capture *via* enhanced root-soil contact to mitigate the effects of drought.

Therefore, this study aimed to investigate morphological (root hair traits) and gene expression mechanisms regulating rhizosheath development in tomato, and the role of ABA in this process. We hypothesized that WT tomato plants will form more rhizosheath under drying stress conditions, relative to an ABA-deficient mutant, because of a root hair phenotype that better allowed sand binding.

## Materials and Methods

### Plant Material and Growth Conditions

Seeds of tomato (*Solanum lycopersicum* L. *cv. Lukullus)* and an ABA-deficient mutant (*notabilis—not*) were obtained from Tomato Genetics Resource Center (University of California, Davis). The *not* mutant has a defect in the gene *Le*NCED1, which encodes a 9-cis-epoxycarotenoid dioxygenase involved in xanthoxin synthesis, a key step in ABA biosynthesis (Thompson et al., 2004).

The seeds were sterilized in a 2.6% sodium hypochlorite solution for 30 min, and then rinsed for 1 h in flowing tap water. The seeds were then sandwiched between two wet Whatman No. 2 filter papers in Petri dishes and placed in the dark to germinate for 5 days. Five-day-old seedlings were transplanted into cylindrical plastic pots (13.5-cm inner diameter, 16-cm height) filled with sieved sand (ø ≤ 0.85 mm), which was collected from Fujian Agricultural and Forestry University botanical beach garden, and watered to different levels: well-watered (WW, 14%), drying stress (DS, 5%); % shows the water content relative to the weight of sand per pot. Pots were covered with black plastic paper material to protect roots from light. They were then transferred to a greenhouse where growth conditions were set at 25 ± 2°C, 60% relative humidity, and light intensity of 150 μmol m^−2^ s^−1^ (Humbeck et al., 1994), supplied by fluorescent lamps fitted with a timer set at 16 h/8 h light/dark photoperiod. All the pots were watered daily to their respective irrigation regimes for 30 days. Three replicates, with two seedlings per pot, were used for each treatment. Five plants with uniform growth were selected for root traits analysis.

### Rhizosheath Quantification

After 30 days of growth, the pots were carefully disassembled, and the root columns were carefully collected and shaken to remove sand not adhered to the root surface, while minimizing disturbance to retain root–sand contact. The roots were detached from the shoots and weighed along with rhizosheath sand. roots were washed in a small jar, and the resulting rhizosheath sand and water in the small jar was dried in a tray at 105°C for 3 days to determine the rhizosheath dry weight. Total root length was determined using an Epson scanner (Epson, Herts, United Kingdom), and the WinRHIZO software (Regent Instruments, Quebec, Canada). The resulting rhizosheath sand and water in the small jar were dried in a tray at 105°C for 3 days to determine the rhizosheath dry weight. Rhizosheath weight per root length was obtained by dividing the total rhizosheath of the individual plant by its corresponding total root length.

### Root Hair Traits Analysis

Three root apical segments (1 mm) excised from each of the 5 experimental plants per genotype/treatment combination were viewed under a Leica stereomicroscope (MZ10F, Germany). Images (JPG format) were captured using a DS-U3 camera (Nikon, Tokyo, Japan) with appropriate magnification (40 ×). The length of a randomly selected root hair from each root apical segment was determined using the Image J software (National Institutes of Health; Supplementary Figure 1). Root hair density was determined as the number of root hairs in each root segments, as previously described (Nestler et al., 2016). The root hair length and density of each individual plant were calculated as the average of the three measurements.

### Root ABA Concentration

In an independent experiment, WT and *not* seedlings were grown under the conditions described above. After ~4 weeks of growth, a root segment (~200 mg root dry weight) was excised, briefly washed to remove adhering sand particles, frozen in liquid nitrogen, freeze dried, and then finely ground in a bead beater (Qiagen, Hilden, Germany) with 3-mm beads at 25 Hz/s for 3 min. Briefly, 200 mg of the sample was placed in a 2-ml tube, then 400 μl ethyl acetate was added, and the mixture was homogenized. Homogenates were centrifuged at 13,000 × g for 10 min at 4°C. Supernatant was transferred into a 2-ml tube. After the second addition of 0.5 ml ethyl acetate with added internal standards (15 ng of ^2^H_6_-ABA) as described by McAdam (2015), the extracts were vacuum-dried at 30°C. The extracts were then dissolved in 70% methanol, vortexed for 20 min, and again centrifuged at 13,000 × g for 10 min at 4°C. The supernatant was carefully transferred to 1.5-ml vials and then injected into the liquid chromatography system.

The samples were analyzed by HPLC-electrospray ionization/MS using Agilent 100 HPLC (Agilent, Santa Clara, CA, United States) coupled with Applied Biosystems Q-TRAP 2000 (Applied Biosystems, California, Foster City, United States). Chromatographic separation was carried out on a 3 μm C18 100 × 2 mm column at 35°C. The mobile phases consisted of solvents A and B (containing 0.1% formic acid and acetonitrile, respectively). Solvent gradient elution mode was programmed as follows: 5–60% B for 0–7.5 min and 60–95% B for 7.5–10 min. The column was then washed with 95% B for 3 min and finally equilibrated with 100% A for 10 min. The injection and flow rates were 2 μl and.4 ml/min, respectively. MS analysis was performed by negative ion mode electrospray ionization (ESI). Optimal conditions were set using the Quantitative Optimization feature (Analyst software) by infusing MS standards with syringe pump while injecting standards into a 200 μl/min flow of 50% solvent A/50% solvent B, and were as follows: cone voltage, 40 V; capillary voltage, 3 kV; temperature, 400°C; desolvation gas flow, 900 L/h; cone gas flow, 50 L/h.

### RNA Extraction

In an independent experiment, WT and *not* seedlings were grown under the conditions described above. Three biological replicates, each containing two plants of each treatment were used. At 4 weeks of growth, about 200 mg root weight of WW and DS plants was harvested, cleaned carefully, immediately frozen in liquid nitrogen, and then stored at −80°C for further analysis. Total RNA was extracted using the TRIzol® kit (Invitrogen, Carlsbad, CA, United States), following the instructions of the manufacturer. Isolated RNA was dissolved in nuclease-free water, and its quality and quantity were estimated by the Agilent Bio-analyzer 2100 system (Agilent Technologies, Santa Clara, CA, United States).

### Illumina RNA Sequencing and Analysis

Equal amounts of RNA samples from WW and DS roots were prepared for RNA-sequencing (RNA-Seq). Sequencing libraries were constructed using NEBNext®Ultra^TM^ RNA Library Prep Kit for Illumina (NEB, Ipswich, MA, USA), following the instructions of the manufacturer. The libraries were sequenced using the BGISEQ-500 sequencer (Beijing Genomics Institute; BGI, Shenzhen, China: Accession no. PRJNA731295). Raw reads obtained from the RNA-Seq were cleaned using SOAPnuk (version 1.5.2). Low-quality reads and those containing adapters or poly-N were eliminated. The resulting high-quality reads were mapped against the *S. lycopersicum* reference genome (ITAG4.0) using HISAT2 (version 2.2.5).

### Differential Gene Expression Analysis and Functional Annotation

To estimate abundance and align reads, a method based on RNA-Seq by Expectation Maximization (RSEM) (Li and Dewey, 2011) was adopted, and bowtie2 (version 2.2.5) was chosen as the alignment method (Mascher et al., 2017). The RSEM method was used to generate expression value matrices, which were normalized as read per million per kilo base (RPKM) by dividing raw reads number multiplied by 1 billion for the transcript length multiplied by total number of mapped reads on each library.

DESeq2 (http://www.biocunductor.org/packages/release/bioc/html/DESeq2.html) was used for differential expression analysis of the four groups (three biological replications per group). The differential expression of transcripts was tested by their significance performing the Fisher's exact test with a *p*-value cutoff ≤ 0.001. The resulting *P-*values were adjusted using the Benjamin and Hochberg approach for dealing with the false discovery rate (FDR) (Benjamini and Hochberg, 1995). The false discovery rate-adjusted *q-*values were calculated using the Benjamini and Hochberg procedure. The log_2_ (fold change) for each gene was calculated. Genes with an adjusted *p-*value ≤ 0.001 [|log_2_ (fold change|) > 1] found by DESeq2 were considered as differentially expressed genes (DEGs).

To assign gene annotations and gene ontology (GO) terms to the predicted tomato genes, a platform based on ITAG4.0 and GO (http://www.geneontology.org/) was used. DEGs were subjected to GO enrichment analysis. The *P*-values from the Fisher's exact test were adjusted using the Benjamini and Hochberg's approach to control the FDR. The GO terms with FDR < 0.001 were considered significantly enriched within the gene set.

### KEGG Enrichment Analysis

Kyoto Encyclopedia of Genes and Genomes is a knowledge database for systematic analysis of gene functions and linking genomic information generated by genome sequencing with higher order functional information. The Kyoto Encyclopedia of Genes and Genomes (KEGG) Orthology Based Annotation System (KOBAS, version 2.0) software was used to analyze statistical enrichment of differentially expressed transcripts in KEGG pathways (http://kobas.cbi.pku.edu.cn/; Xie et al., 2011).

### RT-qPCR Validation

To validate the expression profiles of DEGs obtained from the RNA-Seq analysis, six pairs of primers (Supplementary Table 1) were designed using Primer Express Software (Applied Biosystems, Waltham, MA, United States). To perform RT-qPCR, isolated RNA was reverse transcribed into cDNA using PrimeScript RT Reagent Kit with gDNA Eraser (Takara, Dalian, China), following the instructions of the manufacturer. Each reaction contained a total volume of 10 μl with 1 μl of diluted first strand cDNA, 5 μl of SYBR Green PCR Master Mix (Applied Biosystems, Waltham, MA, United States), and 10 μM of forward and reverse primer. Reactions were performed in Applied Biosystems 7500 Real-Time PCR Systems. The ubiquitin gene was used as an internal control to normalize the expression levels (Czechowski et al., 2005). For each treatment, reactions were performed with three biological replicates pooled from three technical replicates. RT-qPCR results were analyzed using the 2^−Δ*ΔCt*^ method (Livak and Schmittgen, 2001).

### Statistical Analysis

Seven independent experiments of the same experimental design were performed, each comprising five replicates of each genotype × treatment combination. Rhizosheath and root traits were measured in experiments 1 to 5, root ABA concentration in experiment 6, and RNA-sequencing in experiment 7. Root traits (rhizosheath weight, root length and their ratio, root hair length, and density) and root ABA concentration were analyzed by two-way ANOVA at *p* ≤ 0.05 significance level using SPSS (version 1.70). Tukey's test was performed for *post hoc* multiple comparisons within groups. Normality of data was evaluated using homogeneity of variance test. Correlation analysis was performed using Spearman's rank correlation tests (*p* ≤ 0.05; *r*^2^ reported = rho squared), with analysis of covariance performed to determine whether different root traits affected rhizosheath weight differently in the two genotypes. DEGs analysis was performed using DEGseq (version 1.18.0) in Bioconductor package; where genes with 2-fold difference and an adjusted *P*-value of ≤ 0.001 were statistically significant. For RT-qPCR data analysis, Student's *t*-test (*p* ≤ 0.05) in SPSS was applied.

## Results

### Root ABA Concentration Mediates Rhizosheath Size in Drying Sand

**Figure 1:**
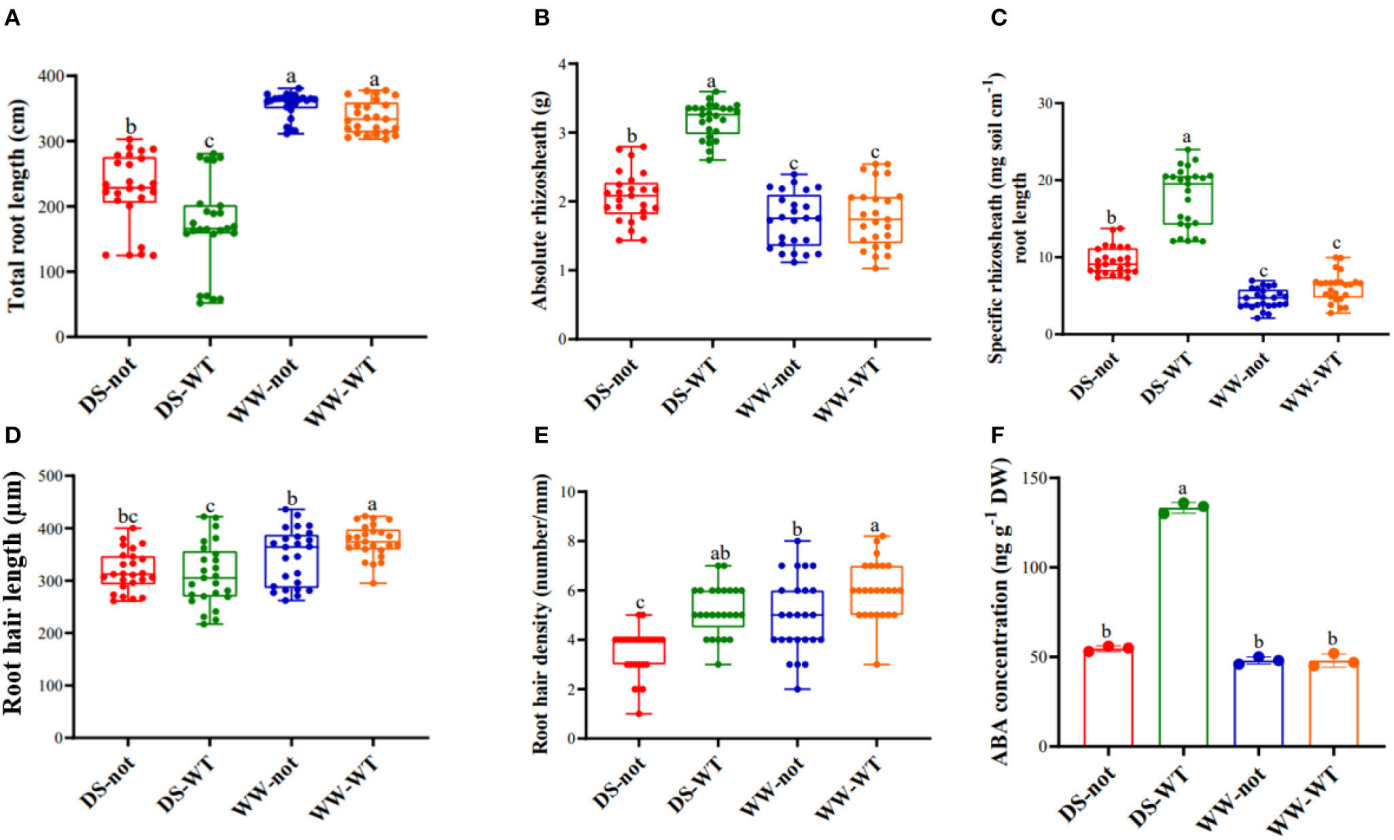
Variation in **(A)** total root length; **(B)** absolute rhizosheath weight; **(C)** specific rhizosheath weight; **(D)** root hair length; **(E)** root hair density, and **(F)** root ABA concentration of WT and *not* plants under contrasting water regime conditions. Data are mean ± s.e. of 25 replicate plants (three replicates for ABA content). Different letters above the bars indicate significant differences at *p* ≤ 0.05. DS, drying stress; WW, well watered; *not, notabilis*; WT, wild type.

While there was no significant genotypic difference in root length under well-watered (WW) conditions, roots of *not* plants were 23% longer than those of WT plants when grown under drying stress conditions (Figure 1A). Although absolute rhizosheath mass was similar in both genotypes under WW conditions, the rhizosheath mass of WT and *not* root systems increased by 1.8- and 1.2-fold, respectively as the sand dried (Figure 1B). When the rhizosheath mass was normalized by root length (the specific rhizosheath mass), there were no significant genotypic differences in the WW plants, but drying stress increased specific rhizosheath mass by 3.1- and 1.9-fold in WT and *not*, respectively (Figure 1C). While root ABA concentrations were similar under WW conditions, drying stress treatment increased root the ABA concentration of WT plants by 2.9-fold but had no effect on *not* (Figure 1F). Thus, ABA accumulation was required for maximal rhizosheath development as the sand dried.

Drying stress also modified root hair traits. WT plants tended (*P* = 0.052) to have longer root hairs, while drying stress decreased root hair length similarly by almost 16% in both genotypes (Figure 1D), as indicated by the no significant genotype × watering treatment interaction (Table 1). In contrast, the root hair density of the WT plants was significantly (*p* < 0.0001) greater than that of *not* (Figure 1E), with the root hair density of both genotypes decreasing similarly by 20% as the sand dried. Thus, drying stress decreased root hair length and root hair density independently of root ABA accumulation.

**Table 1:** Root length, rhizosheath, root hair length and density, and root ABA concentration of *not* and WT plants grown under contrasting water regime conditions, with *P*-values for genotype (G), watering treatment (W), and their interaction (G × W) indicated.

	***P*** **-value**		
	**Genotype**	**Watering**	**G × W**
Total root length (cm)	<0.0001	<0.0001	0.074
Absolute rhizosheath (g)	<0.0001	<0.0001	<0.0001
Specific rhizosheath (mg cm^−1^)	0.002	<0.0001	0.005
Root hair length (μm)	0.052	<0.0001	0.23
Root hair density (number mm^−1^)	<0.0001	<0.0001	0.071
ABA (ng g^−1^ DW)	<0.0001	<0.0001	<0.0001

**Figure 2:**
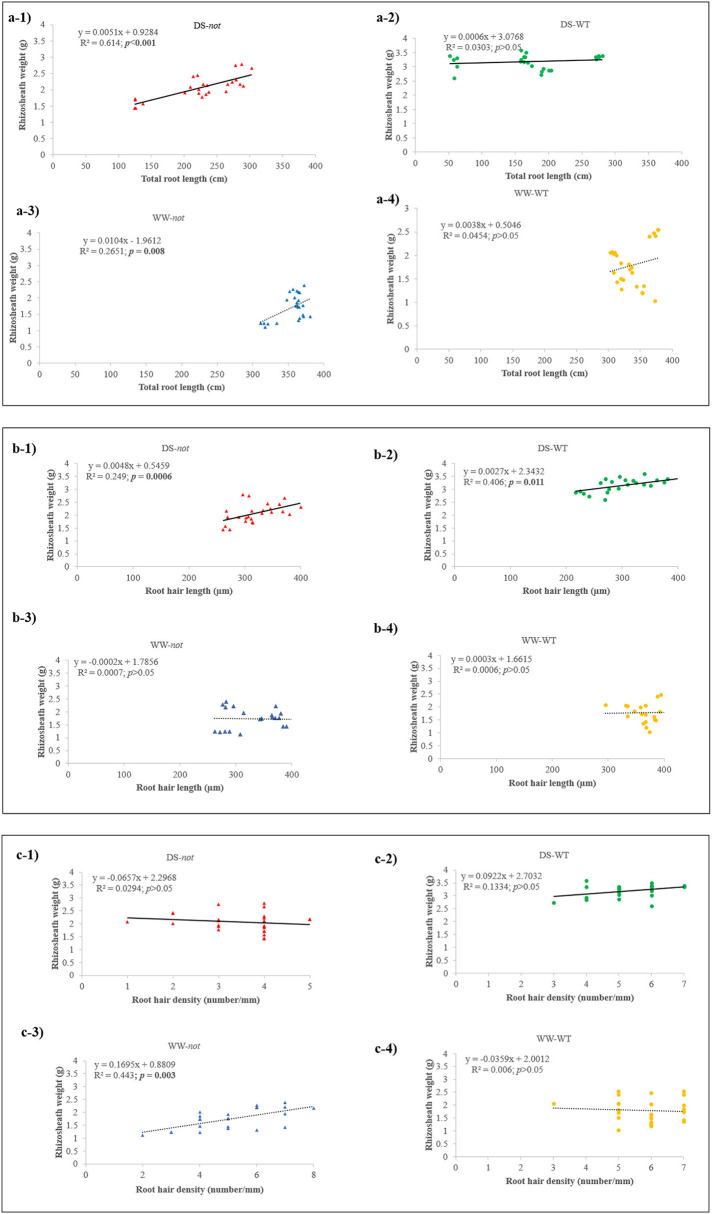
Rhizosheath weight plotted against total root length **(a1–a4)**, root hair length **(b1–b4)**, and root hair density **(c1–c4)**. Each point represents an individual plant, and linear correlations of WT and *not* plants were fitted where significant (*p* ≤ 0.05) *P*-values are shown in bold for plants grown under drying stress (solid lines) and WW (dashed lines) conditions, with *P-*values and *r*^2^ reported. WT (orange circles) and *not* (blue triangles) plants grown under WW conditions; and WT (green circles) and *not* plants (red triangles) grown under drying stress conditions are indicated. Regression lines are fitted to all panels, with *r*^2^ and *P*-values indicated.

Under well-watered conditions, rhizosheath weight tended to increase (*P* = 0.055) with root length in both genotypes (Figure 2A). Under drying stress conditions, rhizosheath weight significantly increased with root length in the *not* plants, but rhizosheath weight was independent of root length in the WT plants. While there was no relationship between rhizosheath weight and root hair length under WW conditions, rhizosheath weight significantly increased with root hair length under drying stress conditions in both genotypes (Figure 2B). In dry sand, the WT plants bound 1.5-fold more sand per unit of root hair length. Generally, rhizosheath weight was not correlated with root hair density, but it significantly increased with root hair density in WW *not* plants (Figure 2C). Taken together, root hair length influenced rhizosheath weight more than root hair density when plants were grown in dry sand.

### RNA-Seq Global Analysis

The 12 libraries were sequenced using the Illumina sequencing platform and 48,120,000–51,090,000 raw reads were obtained. An average of 6.78 G of clean data was obtained from each sample after removing reads of low quality, adaptor contamination and excessively high levels of unknown base N. Approximately 93.09% of clean reads were aligned to the reference genome (Supplementary Table 2). The average alignment of the gene set was 80.98%, and a total of 23,112 genes were detected.

**Figure 3:**
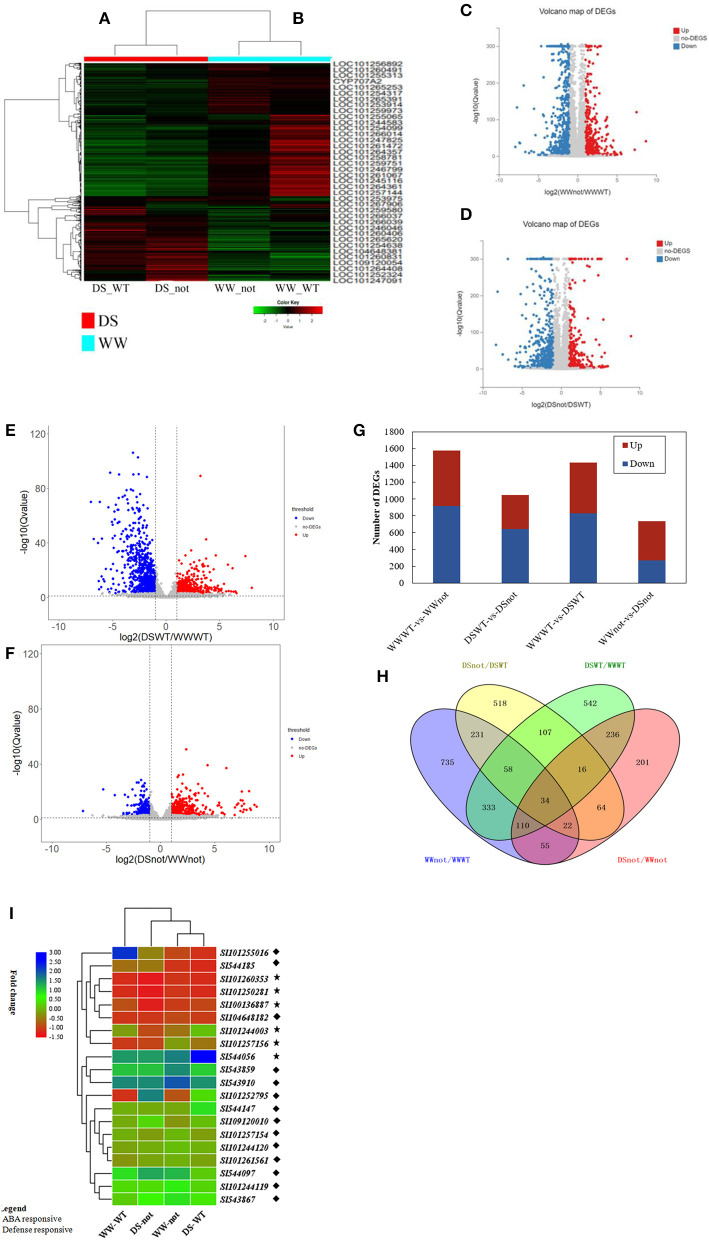
Transcriptional variation in differentially expressed genes of tomato wild-type (WT) and mutant (not) responsive to drying stress and heatmap of DEGs compared under DS-WT and DS-not **(A)**; WW-WT and WW-not **(B)**, based on the expression levels (RPKM+1). Genes in red and green represent highly and lowly expressed genes, respectively. **(C–F)** Volcano plots of the WW-WT/WW-*not*, DS-WT/DS-*not*, DS-WT/WW-WT, and DS-*not/*WW-*not* groups. The X-axis represents the fold change in the difference after conversion to log2, and the Y-axis represents the significance value after conversion to –log10. Red represents upregulated DEGs, blue shows downregulated DEGs, while gray represents non-DEGs; **(G)** graph showing number of up- and downregulated genes; X-axis represents the alignment scheme of DEGs for each group, and the Y-axis represents the corresponding number of DEGs. Red represents the number of upregulated DEGs, and blue represents the number of downregulated DEGs; **(H)** Venn diagram showing the number of DEGS with WW and DS treatments (statistically significant ≥2-fold, *p*-value < 0.05); each circle represents a group of gene sets, and the overlapping region represents common DEGs between different treatments; **(I)** Heatmap of DEGs responsive to ABA and defense, at *p*-value < 0.001 and log2 (fold change) >1.

Differential gene expression was calculated using a Poisson distribution model. Global gene expression profiles under two different water regime conditions are shown on a heatmap by comparing WW-WT/WW-*not*, and DS-WT/DS-*not* (Figures 3A,B). The most highly differential expressed genes were visualized using volcano plot. The log2 values of WW-WT/WW-*not*, DS-WT/DS-*not*, DS-WT/WW-WT, and DS-*not/*WW-*not* were plotted against –log10 (Figures 3C–F). Graphical representation of upregulated and downregulated genes is shown in Figure 3G. With the WW-WT/WW-*not* treatments, a total of 1,578 DEGs, including 916 upregulated and 662 downregulated genes, were identified. Comparatively, with DS-WT/DS-*not* treatments, a total 1,050 DEGs, including 642 upregulated and 408 downregulated genes, were identified.

### DEGs Responsive to ABA and Drought

The differentially expressed genes (DEGs) were assigned various functions using GO terms. In both DS-WT and DS-*not*, the most enriched GO terms were ABA and defense responses (Supplementary Figure 3B). Under drought stress conditions, 26 upregulated and 14 downregulated DEGs were related to ABA signaling, whereas 20 upregulated and 24 downregulated DEGs were found for defense responses (Figure 3I, Supplementary Datasets 1, 2). Several water deprivation-responsive genes, such as APETALA2-like ethylene responsive transcription factor/AP2-like ERF TF (At1g16060), and dehydrins, such as TAS 14 peptide (LOC_544056), were upregulated by the ABA signaling pathway. In addition, ABA 8′-hydroxylase (LOC_100136887), which encodes an enzyme responsible for ABA catabolism, was also upregulated by ABA. On the other hand, drought upregulated several defense-related genes, such as lysine M domain receptor-like kinase (LOC_101260353), pathogenesis-related proteins, protein TIFY 5A-like (LOC_101255016), and tyrosine- and lysine-rich proteins, but downregulated protein phosphatase 2C (Supplementary Datasets 1, 2).

### Gene Ontology Functional Analysis of DEGs

To identify the biological functions, the differentially expressed genes (DEGs) were used to perform gene ontology (GO) analysis based on sequence homologies. Comparing the WW-WT/WW-*not* group, the four main GO categories in the biological process were cellular processes, metabolic processes, response to stimulus, and biological regulation. In the cellular component, the four main GO terms were cell, cell part, organelle, and membrane; while in the molecular function, binding, catalytic activity, transcription regulator activity, and transporter activity were the four main GO categories (Supplementary Figure 2A). Comparing the DS-WT/DS-*not* groups, similar GO terms in biological processes, cellular components, and molecular functions were identified (Supplementary Figure 2B).

To further validate the number of genes involved in the GO enrichment analysis, a bubble chart graph was designed. Rich ratio was calculated as follows: rich ratio = term candidate gene number/term gene number. In the WW-WT/WW-*not* group, the two main gene sets of GO terms in the biological process were 72 genes in defense response and 55 genes responsive to abscisic acid; in cellular component were 313 genes in integral membrane protein and 189 genes in the plasma membrane; and in molecular function were 161 genes in DNA-binding transcription activity and 98 genes in sequence-specific DNA binding (Supplementary Figure 3A). On the other hand, in the DS-WT/DS-*not* group, the main gene sets of GO terms in the biological process were 44 genes in defense response and 40 genes responsive to abscisic acid; in cellular component were 229 genes in integral membrane protein and 119 genes in plasma membrane; and in molecular function were 38 genes in DNA-binding transcription activity and 98 genes involved in heme binding (Supplementary Figure 3B).

### KEGG Enrichment Analysis of DEGs of Tomato Wild-Type and Mutant *Not* Under Control and Drought Stress Conditions

To further study the biological pathways of differentially expressed genes (DEGs) triggered by water stress, the DEGs were annotated by blast analysis against the KEGG database. Genes that were up- or downregulated with WW and DS treatments in both WW and mutant tomatoes were subjected to KEGG enrichment analysis (Supplementary Figure 4). A total of 1,578 DEGs identified above were significantly enriched in 44 KEGG pathways in the WW-WT/WW-*not* and DS-WT/DS-*not* group comparison. The three major enriched gene KEGG pathways were “Signal transduction,” “Global and overview maps,” and “Immune system” in both groups.

### Root Transcriptional Expression of ABA- and Drought-Related Genes

RNA-seq results revealed multiple genes involved in ABA and drought signaling pathways. Of these, six candidate ABA- and drought-responsive genes that were highly differentially expressed in the two genotypes under WW and drying stress conditions were selected for confirmation by RT-qPCR. They were involved in different pathways of plant defense, namely, the pathogenesis-related protein 4 (PR 4) and protein TIFY 5A-like, and ABA signaling pathways, such as LysM domain receptor-like kinase (Lyk14), APETALA2-like ethylene-responsive transcription factor (AP2-like ER TF), abscisic acid 8′-hydroxylase, protein phosphatase 2C (PP 2C), and the TAS 14 peptide.

**Figure 4:**
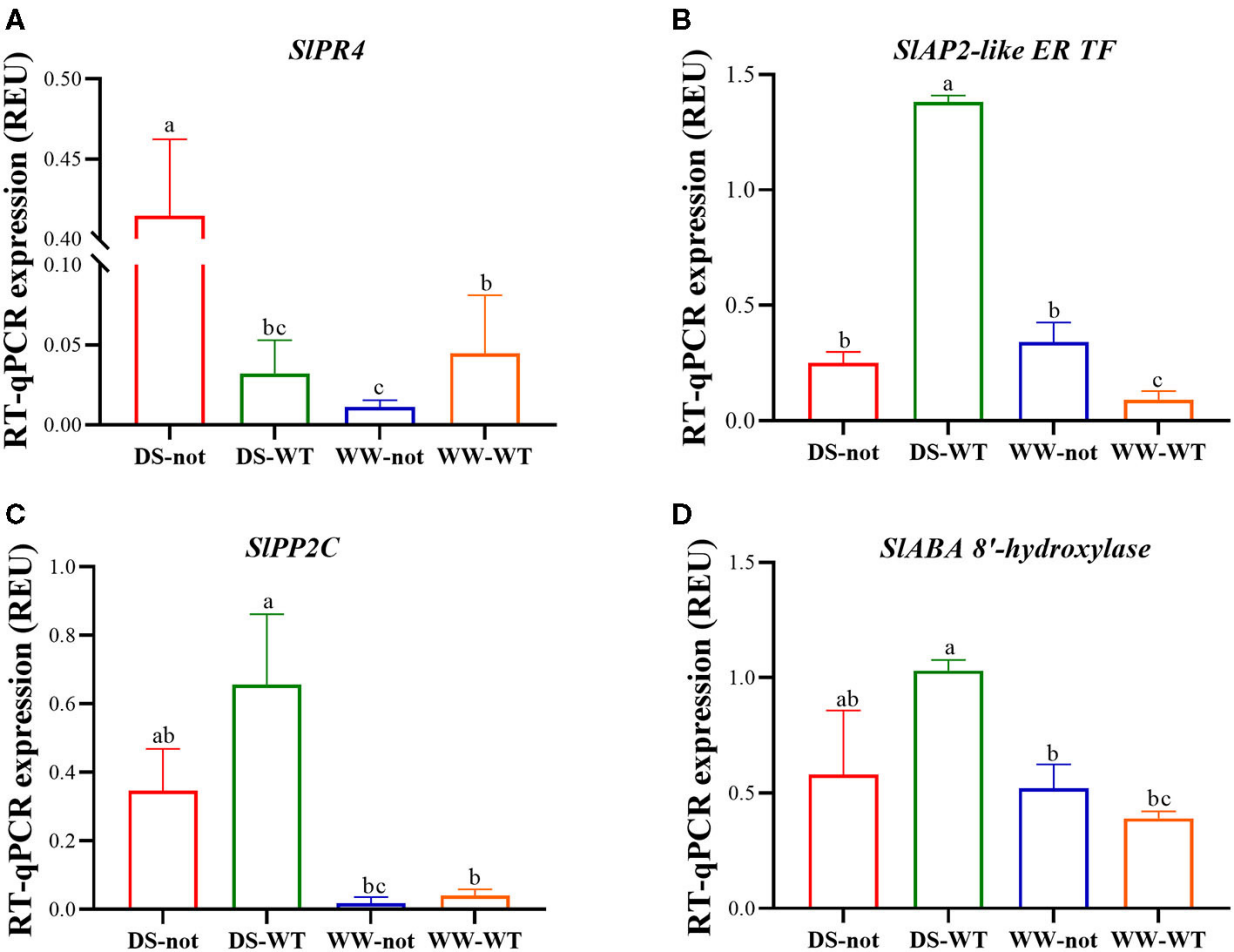
Root expression of genes encoding the *Solanum lycopersicum* (*Sl*) pathogenesis-related protein 4 (*SlPR 4*) **(A)** and APETALA2-like ethylene-responsive transcription factor (*SlAP2-like ER TF*) **(B)**, protein phosphatase 2C (*SlPP 2C*) **(C)**, abscisic acid 8′-hydroxylase (*SlABA 8*′*-hydroxylase*) **(D)** based on RT-qPCR data. Columns represent the relative expression levels of genes with different treatments. Bars on top of the columns represent the mean ± standard error (s.e.) of three biological replicates. The ubiquitin gene was used as an internal control to normalize the expression levels. Significantly different (*p* ≤ 0.05) expression levels with different treatments are indicated with different letters.

Root gene expression of the pathogenesis-related protein 4 (PR 4) was low under well-watered conditions and in droughted WT plants, but increased more than 4-fold in droughted *not* plants (Figure 4A). While there were no genotypic differences in root expression profiles of AP2-like ER TF under WW conditions, drying stress increased its expression by 5.5-fold in WT roots but had no effect on *not* roots (Figure 4B). While gene expression of protein phosphatase 2C (PP 2C) was similar in both genotypes under well-watered conditions, drying stress significantly increased its expression by 1.9-fold in WT roots with an attenuated response in *not* roots (Figure 4C). Drying stress treatment increased the gene expression of ABA 8′-hydroxylase in WT roots by almost 1.7-fold, but had no significant effect on *not* roots (Figure 4D). Taken together, drought-induced ABA accumulation upregulated ABA-related and defense genes, which may have modulated root traits favoring soil adherence to the roots.

## Discussion

ABA-mediated processes help plants adapt to drought by modifying root system architecture, physiological responses, and expression of stress-responsive genes (Cutler et al., 2010). Nonetheless, the role of ABA in mediating drought-induced rhizosheath formation in crop species is still unclear (Zhang et al., 2020). Comparing the drought stress response of WT and ABA-deficient tomato plants revealed that ABA accumulation, to some extent, inhibited root elongation in dry sand, but stimulated rhizosheath formation, in part by attenuating decreases in root hair density and possibly by modifying stress-responsive gene expression. While root hairs seem important in physically enmeshing soil particles (Koebernick et al., 2017; De Baets et al., 2020), the greater rhizosheath weight of the WT plants per unit of root hair length (Figure 2B) suggests that ABA mediates changes in exudate chemistry that determine rhizosheath formation as the sand dries.

Whereas previous studies demonstrated root growth (volume, surface area, and length) of *notabilis* was substantially less than that of WT plants when grown in loamy sand (Tracy et al., 2015), here genotypic effects on root length seemed to depend on soil water status. While there were no genotypic differences in root length under WW conditions, the *not* plants had 23% longer root length than WT plants when grown under drying stress conditions (Figure 1A), contrary to experiments showing that ABA is required to maintain primary root elongation of maize plants under drying stress conditions (Spollen et al., 2000) and lateral root development of tomato (Tracy et al., 2015). Nevertheless, the *not* plants grown *in vitro* had more and longer lateral roots than the WT plants (Belimov et al., 2014), suggesting that in some substrates, ABA may actually inhibit root growth. Under drying stress condition, roots of the *not* plants did not accumulate ABA (Figure 1F) in contrast to WT plants, suggesting that drought-induced root ABA accumulation restricted the root length of the WT plants. Indeed, high concentrations (>1 μM) of exogenous ABA can inhibit root growth (Rowe et al., 2016), thus a certain threshold of ABA accumulation is required for root growth beyond which growth is arrested.

In contrast, drying stress promoted the rhizosheath formation of both tomato genotypes as in other crop species (Watt et al., 1994; Albalasmeh and Ghezzehei, 2014). The WT plants having greater rhizosheath than the *not* plants (Figure 1B) indicates that ABA is needed for maximal rhizosheath development as the sand dries. Both genotypes had similar rhizosheath development under WW conditions consistent with their similar root ABA concentration under these conditions. Likewise, the ABA-deficient barley mutant *Az34* had restricted rhizosheath development independent of soil water status, associated with its lower root ABA concentration (Zhang et al., 2021). Nevertheless, the mechanisms by which root ABA accumulation promote rhizosheath formation require further investigation.

Growth of root hairs is highly plastic and is regulated by a wide range of both endogenous and environmental inputs (Bustos et al., 2010; Zhu et al., 2010; Chandrika et al., 2013). Root hairs greatly increase root-soil contact to facilitate nutrient and water absorption (Haling et al., 2013), implying that the length and/or density of root hairs formed helps plants adapt to stressful conditions. While rhizosheath weight was directly correlated with root hair length in wheat (Delhaize et al., 2012), these traits were weakly associated in an array of barley genotypes (George et al., 2014), and no association was observed in diverse chickpea genotypes (Pang et al., 2017). Whereas drying stress increased root hair length of cotton (Xiao et al., 2020) and orange (Zhang et al., 2019), root hair length of both tomato genotypes decreased under drying stress conditions (Figure 1D, Table 1). Nevertheless, the roots of the WT plants held more sand per unit of root hair length as the sand dried (Figure 2B), which may help acquire soil moisture by increasing root-soil contact in sandy soils (North and Nobel, 1997; Smith et al., 2011).

Although the WT plants had longer and more numerous root hairs than *not* under WW conditions (Figures 1D,E), this did not affect rhizosheath development. Drying the sand substantially decreased the root hair density of tomato (Figure 1E), in contrast to the increase in other species (Liu et al., 2019; Zhang et al., 2019). Under drying stress conditions, the root hair density of the WT plants was 33% higher than that of the *not* plants, consistent with the genotypic differences in rhizosheath weight. While this ABA-mediated attenuation of the decline in root hair density with sand drying is associated with greater rhizosheath development of the WT plants, it is interesting that rhizosheath weight was highly correlated with root hair length in both genotypes under drying stress conditions (Figure 2B), with the WT plants binding 1.5-fold more sand at the same root hair length. This may also imply differences in root hair chemistry affecting binding of sand particles (Watt et al., 1994; Haling et al., 2010, 2014; George et al., 2014; Pang et al., 2017).

Transcriptome analysis revealed that drought-related and ABA signaling pathway genes were differentially regulated under drought. TFs serve as master regulators of cellular processes by regulating downstream genes related to drought tolerance (Gahlaut et al., 2016; Joshi et al., 2016), and AP2/ERFs can regulate drought stress responses in *S. lycopersicum* (Wu et al., 2008). Drying stress upregulated root SlERF5 expression, while overexpressing SlERF5 increased drought stress tolerance by increasing leaf relative water content (Pan et al., 2012). Upregulation ofAP2/ERF transcription factor genes in the roots in dry sand was ABA-dependent (Figure 4B) and may be important in ABA-mediated rhizosheath development, by affecting as yet unidentified mechanisms.

Although SlERF5 overexpression in tomato stimulated foliar expression of pathogenesis-related (PR) defense genes (Li et al., 2011), the root PR4 gene expression under drying stress conditions showed an opposite response to AP2/ERF transcription factor gene expression (cf. Figures 4A,B). Upregulation of the PR4 expression in the *not* roots in drying stress (Figure 4A) was independent of both ABA and AP2/ERF transcription factor gene expressions, which increased only in WT roots under drying stress conditions. In contrast, both exogenous ABA and drought upregulated the OsPR4 expression in rice leaves (Wang et al., 2011), with relatively low expression levels in the roots compared with above-ground tissues. Notwithstanding possible differential regulation of PR4 genes in rice and tomato, enhanced root PR4 gene expression in *not* may enhance resistance against biotic stresses, at least under drying stress conditions. In support, *not* was less susceptible to root-knot nematode infection, associated with its higher constitutive and nematode-induced expression of the defense-related gene plant defense factor (PDF) and the proteinase inhibitors PI-1 and PI-2 (Xu et al., 2019). Whether PR4 is associated with rhizosheath formation in drying stress conditions requires rhizosheath measurements in genotypes under- or over-expressing PR4 (Wang et al., 2011).

In plants, reversible protein phosphorylation mediated by protein phosphatases and kinases is an important adaptive cellular response maintaining phospho-regulation under normal and stressful growth conditions. Protein phosphatases, especially Clade A of PP 2C, have been implicated in *Arabidopsis* and rice signaling pathways triggered by stress such as drought (Singh et al., 2010; He et al., 2019). Drought-induced upregulation of the root PP 2C gene expression in the WT roots was consistent with increased root ABA concentration (cf. Figures 1F, 4C), whereas high variability of the root PP 2C gene expression in the *not* roots was inconsistent with the lack of root ABA accumulation. Taken together, the ABA-dependent expression of PP 2C genes in roots may play important roles in regulating root growth response to drying stress.

Although genotypic and drought-induced differences in root ABA concentrations were consistent with the expected phenotypes based on knowledge of NCED gene expression (Thompson et al., 2000), stress-induced ABA accumulation can also be modulated by ABA conjugation or oxidation, especially ABA 8′-hydroxylation catalyzed by CYP707A proteins (Kushiro et al., 2004; Saito et al., 2004). Although drying stress treatment stimulated the expression of an ABA 8′-hydroxylase (CYP707A1) gene in the WT roots (Figure 4D), implying that CYP707A1 might be involved in ABA catabolism in these tomato roots, root ABA concentration still increased in association with root length inhibition compared with the *not* plants. Thus, ABA biosynthesis was greater than ABA metabolism in the WT roots, although the import of ABA from the shoot (McAdam et al., 2016) might provide an additional source of ABA.

In conclusion, gene expression responses to drying stress induced root ABA accumulation and expression of associated signaling genes in the WT plants. Although these responses diminished root exploration of the dry sand compared with the *not* plants, they greatly promoted rhizosheath formation. This ABA-dependent stimulation of rhizosheath development under drying stress conditions was independent of root hair length and density, suggesting that future studies should focus on the effects of root ABA status on exudate chemistry.

## Data Availability Statement

The datasets presented in this study can be found in online repositories. The names of the repository/repositories and accession number(s) can be found at: PRJNA731295, https://www.ncbi.nlm.nih.gov/bioproject/PRJNA731295.

## Author Contributions

XW and ID conceived the study and supervised the whole research. JK performed most of the experiments, analyzed the data, and wrote the manuscript. MA, ZQ, XW, and RY reviewed the study. ID critically reviewed and revised the study. All authors contributed to the article and approved the submitted version.

## Conflict of Interest

The authors declare that the research was conducted in the absence of any commercial or financial relationships that could be construed as a potential conflict of interest.

## Publisher's Note

All claims expressed in this article are solely those of the authors and do not necessarily represent those of their affiliated organizations, or those of the publisher, the editors and the reviewers. Any product that may be evaluated in this article, or claim that may be made by its manufacturer, is not guaranteed or endorsed by the publisher.

## Supplementary Material

Supplementary Figure 1 Rhizosheath formation of **(A)** *notabilis* and **(B)** wild-type plants growing under drying stress conditions; and **(C)** *notabilis* and **(D)** wild-type plants growing under well-watered conditions, **(E)** root hair analysis as determined using the Image J software, and **(F)** root length as determined by the WinRhizo program.

Supplementary Figure 2 GO enrichment analysis of the DEGs in two different group comparisons.

Supplementary Figure 3 GO enrichment analysis of DEGs represented by bubble chart.

Supplementary Figure 4 KEGG enrichment analysis of the two-group comparison.

Supplementary Dataset 1 Differentially expressed genes (DEGs) related to ABA response (Excel file separately attached).

Supplementary Dataset 2 Differentially expressed genes (DEGs) related to defense response (Excel file separately attached).

Supplementary Table 1 Primers used for RT-qPCR expression analysis.

Supplementary Table 2 Summary of read numbers and alignment obtained from RNA sequence of drought-stressed and control samples.

Supplementary Table 3 Fold changes in gene expression in rhizosheath-bound roots under drying stress compared to well-watered conditions in two tomato genotypes (*Lukullus* and *notabilis*) determined by RNA-seq and RT-qPCR.
