# Supplementary material for: Abscisic Acid Mediates Drought-Enhanced Rhizosheath Formation in Tomato
Source: Front Plant Sci. 2021 Jul 23;12:658787. doi: 10.3389/fpls.2021.658787 (PMC8378331; doi:10.3389/fpls.2021.658787)
Supplement: Supplementary Figure 1 — Rhizosheath formation of (A) notabilis and (B) wild-type plants growing under drying stress conditions; and (C) notabilis and (D) wild-type plants growing under well-watered conditions, (E) root hair analysis as determined using the Image J software, and (F) root length as determined by the WinRhizo program. [file Data_Sheet_1.ZIP › Supplementary Files/Frontiers Supplementary_Material.doc]

**Supplementary Data**

**Fig. S1** Rhizosheath formation in *notabilis* (a), wild-type (b) under DS/soil drying; WW/well-watered *notabilis* (c), and wild-type (d); and root hair analysis as determined using Image J software (e).

**Fig. S2** GO enrichment analysis of the DEGs in two different group comparisons. a) Classification of the enriched GO terms under the WW-WT-vs-WW-*not* groups; b) classification of the enriched GO terms under the DS-WT-vs-DS-*not* groups. X-axis represents the number of genes annotated to the GO entry, and the Y-axis represents the GO functional classification.

**Fig. S3** GO enrichment analysis of DEGs represented by bubble chart**.** X-axis is the enrichment ratio (the ratio of the number of genes annotated to an entry in the selected gene set to the total number of genes annotated to the entry in the species). The size of the bubble represents the number of DEGs annotated to a GO term, while the color represents the enriched Q value; the deeper the color, the smaller the Q value.

**Fig. S4** KEGG enrichment analysis of the two group comparison. a) WW-WT-vs-WW-*not* as control groups b) DS-WT-vs-DS-*not* as treatment groups. X-axis represents the number of genes annotated to a category of KEGG pathway, and Y-axis represents the category of KEGG pathway.

**Table S1** Primers used for RT-qPCR expression analysis.

| Primer | Sequence (5′ → 3′) |
| --- | --- |
| Lyk14-F | GCTGTAGATACTGCAAGGGGT |
| Lyk14-R | CCAGGCCAAAATCTGCAACC |
| AP2-like ER TF-F | CGTGACCACGACTTCATCCT |
| AP2-like ER TF-R | GATCCGGGTCGGGTAATTCG |
| ABA 8'-hydroxylase CYP707A1-F | GTCCCATTGGGTGGACTAGC |
| ABA 8'-hydroxylase CYP707A1-R | GGTGGGGTTTGGGGTTGATA |
| Protein phosphatase 2C 24-F | GCGAGCCAGAAGTGACGATA |
| Protein phosphatase 2C 24-R | CGCCTAGCAAGGGCTAGTTT |
| pathogenesis-related protein P4-F | GGTGTCCGAGAGGCCAGAC |
| pathogenesis-related protein P4-R | GAGTTGCGCCAGACTACTTGA |
| protein TIFY 5A-like-F | TCGTTGTATCGTTTTGCTCTGT |
| protein TIFY 5A-like-R | GAGCGAGATATCCGTGCCTT |
| Ubiquitin3-F | GAAGAAGAAGACCTACACCAAGCC |
| Ubiquitin3-R | CACTCCTTACGAAGCCTCTGAAC |

F- Forward; R- reverse

**Table S2** Root length, rhizosheath, root hair length and density, and root ABA concentration of *not* and WT plants grown under contrasting water regimes, with *P* values for genotype (G), watering treatment (W) and their interaction (G×W) indicated.

| *P*-value  Genotype Watering G×W | | | |
| --- | --- | --- | --- |
| Total root length (cm)  Absolute rhizosheath (g) | < 0.0001  < 0.0001 | < 0.0001  < 0.0001 | 0.074  < 0.0001 |
| Specific rhizosheath (mg cm^-1^) | 0.002 | <0.0001 | 0.005 |
| Root hair length (µm) | 0.052 | < 0.0001 | 0.23 |
| Root hair density (number mm^-1^)  ABA (ng g^-1^ DW) | <0.0001  <0.0001 | < 0.0001  <0.0001 | 0.071  <0.0001 |

**Table S3** Summary of read numbers and alignment obtained from RNA sequence of drought-stressed and WT samples.

| Sample | Total Raw Reads (M) | Total Clean Reads (M) | Total Clean Bases(Gb) | Clean Reads Q20 (%) | Clean Reads  Q30 (%) | Clean Reads  Ratio (%) |
| --- | --- | --- | --- | --- | --- | --- |
| DS-WT1 | 48.52 | 44.68 | 6.7 | 96.87 | 88.68 | 92.08 |
| DS-WT2 | 50.83 | 46.31 | 6.95 | 96.89 | 88.78 | 91.11 |
| DS-WT3 | 51.09 | 46.8 | 7.02 | 96.94 | 88.88 | 91.61 |
| DS-not1 | 49.08 | 44.83 | 6.72 | 96.84 | 88.65 | 91.34 |
| DS-not2 | 49.08 | 44.97 | 6.75 | 96.86 | 88.7 | 91.64 |
| DS-not3 | 49.59 | 44.67 | 6.7 | 96.89 | 88.77 | 90.08 |
| WW-WT1 | 48.98 | 44.87 | 6.73 | 97.08 | 89.32 | 91.61 |
| WW-WT2 | 49.08 | 44.64 | 6.7 | 96.93 | 88.91 | 90.95 |
| WW-WT3 | 48.12 | 44.35 | 6.65 | 96.91 | 88.81 | 92.17 |
| WW-not1 | 49.08 | 45.21 | 6.78 | 97.06 | 89.25 | 92.11 |
| WW-not2 | 49.08 | 44.82 | 6.72 | 97.03 | 89.12 | 91.32 |
| WW-not3 | 50.83 | 45.95 | 6.89 | 96.91 | 88.86 | 90.39 |

**Table S4** Fold changes in gene expression in rhizosheath-bound roots under soil drying compared to well-watered conditions in two tomato genotypes (wild-type and *notabilis*) determined by RNA-seq and RT-qPCR.

|  |  | RNA-seq (log2 Ratio) | | | | RT-qPCR (log2 Ratio) | | | | |
| --- | --- | --- | --- | --- | --- | --- | --- | --- | --- | --- |
| Gene ID | ^a^Annotation | DS-*not* | DS-WT | WW-*not* | WW-WT |  | DS-*not* | DS-WT | WW-*not* | WW-WT |
| 101260353 | lysM domain receptor-like kinase | 0.01 | 0.3 | 0.03 | 0.01 |  | 0.12 | 1.04 | 0.75 | 0.13^b^ |
| 101250281 | AP2-like ethylene-responsive transcription factor At1g16060 | 0.06 | 0.26 | 0.05 | 0.04 |  | 0.25 | 1.38 | 0.34 | 0.09^b^ |
| 100136887 | ABA 8'-hydroxylase CYP707A1 | 0.09 | 0.98 | 0.12 | 0.53 |  | 0.58 | 1.03 | 0.52 | 0.39^b^ |
| 101244003 | protein phosphatase 2C | 0.14 | 0.27 | 0.06 | 0.09 |  | 0.35 | 0.66 | 0.02 | 0.04^b^ |
| 544185 | pathogenesis-related protein P4 | 2.77 | 1.4 | 1.48 | 1.42 |  | 0.41 | 0.03 | 0.01 | 0.04^b^ |
| 101255016 | protein TIFY 5A-like | 0.75 | 3.14 | 0.27 | 0.5 |  | 0.14 | 0.76 | 0.04 | 0.03^b^ |

Abbreviations: DS, soil drying; WW, well-watered; WT, wild-type; *not*, *notabilis*.

^a^Annotation based on NCBI nr. ^b^Fold change with statistical significance (*P*<0.05).
